# Colloid Cyst Presenting With Severe Headache and Bilateral Leg Weakness: Case Report and Review

**DOI:** 10.7759/cureus.49347

**Published:** 2023-11-24

**Authors:** Arsam Nadeem, James Espinosa, Alan Lucerna

**Affiliations:** 1 Emergency Medicine, Jefferson Health, Stratford, USA

**Keywords:** foramen of monro cyst, leg weakness from colloid cyst, headache from colloid cyst, obstructive hydrocephalus, hydrocephalus, third ventricle cyst, colloid cyst

## Abstract

Colloid cysts are benign growths commonly found in the third ventricle at the foramen of Monro. They can be asymptomatic or present with a variety of symptoms, including headaches, diplopia, memory problems, psychosis, urinary incontinence, and ataxia. Obstructive hydrocephalus from a colloid cyst is caused by a blockage of cerebral spinal fluid (CSF) from the lateral ventricles at the foramen of Monro. Colloid cysts have rarely been reported to cause sudden death. Here we present the case of a 32-year-old female with a known history of a colloid cyst who presented with a headache and transient weakness in her lower extremities noted while climbing stairs.

## Introduction

Colloid cysts are composed of an epithelial lining with goblet cells and are filled with proteinaceous material. They represent a rare class of intracranial growth and are considered to be a developmental malformation in the brain and not true neoplasms [1]. Colloid cysts account for less than 1% of all primary brain tumors [2]. Although rare, they can cause significant neurological dysfunction. Here, the case is of a 32-year-old female with a known history of a colloid cyst who presented with a headache and transient weakness in her lower extremities. This case was presented as a poster at the Rowan University Research Day in Stratford, New Jersey, on May 4, 2023.

## Case presentation

A 32-year-old female with a known history of a colloid cyst presented to the emergency department (ED) with a complaint of a headache. An hour prior to her arrival, she came home from work and, while climbing stairs, felt a sudden sharp headache along with an associated weakness of her lower extremities. She was subsequently moved to the couch with the help of her grandmother. As she described it, “It felt like my legs were cement and my grandmother had to help me move.” The emergency medical service was called, and the patient was promptly brought to the ED for further evaluation. The patient's leg weakness had resolved by the time of her arrival at the ED, and her headache had improved. She denied loss of consciousness, bowel or bladder incontinence, or seizure-like activity. She reported similar sudden, brief, and intense headaches in the month prior to the ED presentation, without any other symptoms. The patient's social history was positive for social alcohol use and negative for tobacco or illicit drug use.

On presentation, her vital signs were as follows: blood pressure 127/56 mmHg, heart rate 87 beats per minute, respiratory rate 18 breaths per minute, temperature 99 degrees Fahrenheit orally, and a pulse oximetry of 99% O2 saturation on room air.

The patient's physical examination revealed a non-toxic, well-appearing female with no focal neurological deficits. Her National Institutes of Health Stroke Scale score was 0. She was alert and oriented to person, place, and time. Her cranial nerves were grossly intact, and she had full strength and sensation in all extremities.

A chest x-ray showed no acute findings. An electrocardiogram (ECG) showed normal sinus rhythm at 84 beats per minute without acute ST segment or T wave changes. A complete blood count and basic metabolic panel were unremarkable. Her urinalysis was noted to have 2+ blood but was otherwise negative.

Computed tomography (CT) of the brain showed an 11-mm colloid cyst at the foramen of Monro, causing moderate to severe obstructive hydrocephalus of the lateral ventricles bilaterally. Computed tomography angiography (CTA) of the brain showed no stenosis or occlusion of the vessels.

**Figure 1:**
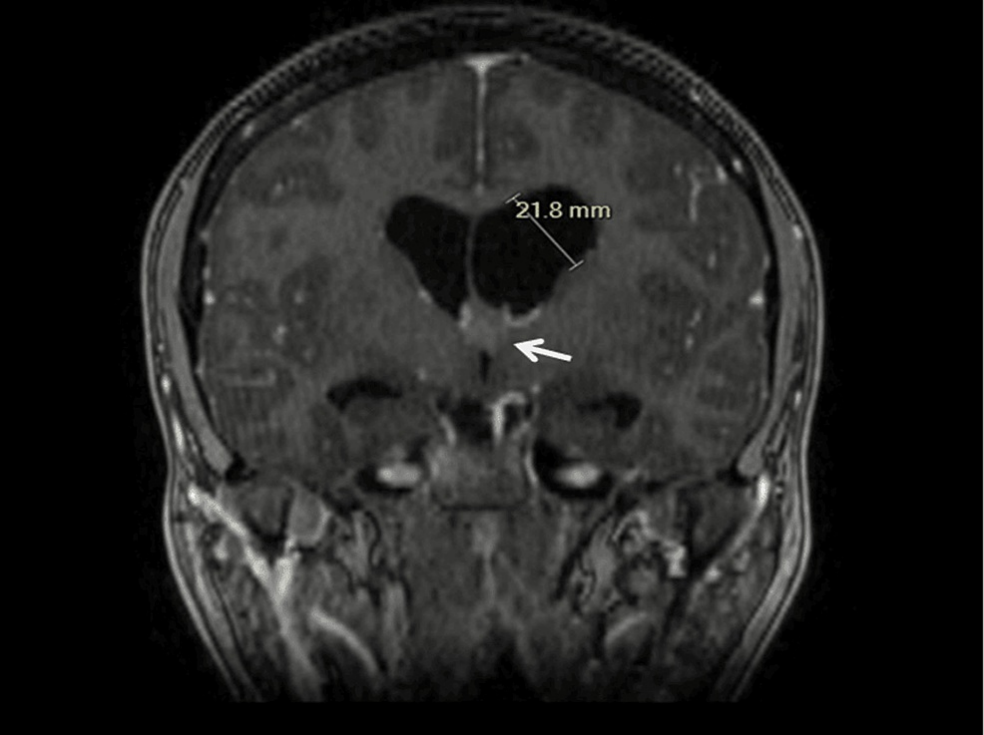
MRI showing an obstructive hydrocephalus measuring 21.8 mm (labelled). A colloid cyst measuring 9 mm is present (arrow).

Magnetic resonance imaging (MRI) of the brain was also obtained, which showed a colloid cyst of the 3rd ventricle causing obstructive hydrocephalus. The MRI characterized the cyst as 9 x 9 x 9 mm in size (Figure 1).

The patient was admitted to the neurological intensive care unit (NICU) for serial neurological checks. The patient was evaluated by neurosurgery and underwent endoscopic colloid cyst removal with the placement of an external ventricular drain (EVD). On hospital day 10, the EVD was removed, and a ventriculoperitoneal shunt was placed. The following day, the patient was discharged home with out-patient neurosurgical follow-up.

## Discussion

Definition and epidemiology

Colloid cysts of the third ventricle are a type of intracranial tumor [1]. They are considered to be rare, accounting for less than 1% of intracranial tumors [2]. They are the most common of the third ventricle tumors. The age range is three weeks to 72 years of age [3]. Most patients are diagnosed between the 3rd and 5th decades of life [1]. The incidence is estimated to be 3.2 cases per 100,000 population per year [3].

Histopathology

Colloid cysts have a fibrous outer capsule with an inner lining of a single layer of epithelial cells and goblet cells. The epithelial cells can be squamous, cuboidal, or low-columnar. The goblet cells secrete a protein-rich material [4]. They lack malignant transformation potential [3].

The origin of a colloid cyst has been proposed to be a possible human remnant of sensory cells in lower vertebrates. The theory holds that these cells, which are epithelial in origin, slowly grow in size as they accumulate secretory debris [1]. Another theory is the development of pouch-like evagination of the diencephalic roof during embryologic development [5].

Pathophysiology

Most cases of hydrocephalus are in the category of normal-pressure hydrocephalus, where the cerebral spinal fluid (CSF) is able to communicate with all parts of the brain. Although histologically benign, a colloid cyst at the foramen of Monro (third ventricle) can cause obstructive hydrocephalus [3]. Obstructive hydrocephalus of the third ventricle can cause alterations in mental status and memory, loss of bladder control and weakness, and ataxia of the lower extremities [1]. Lower extremity weakness appears to be a result of pressure on the lateral wall of the third ventricle, with a subsequent pressure effect on the pyramidal fibers to the legs, which are adjacent to the wall of the third ventricle [6].

The common symptom of headache in hydrocephalus related to a colloid cyst is generally explained as the direct effect of intermittent obstruction of CSF flow. A ball-valve mechanism may explain the intermittent nature of the headache as well as the possible relationship of the headache to body position [1].

Presentation

The most common chief complaint at presentation is a headache. The headache is episodic, with brief episodes of severe headache that may last seconds to minutes. Position changes can alleviate the headaches. Some patients will experience a syncopal episode at the peak of the headache [1].

However, patients can present with a variety of neurologic symptoms based on the degree of ventriculomegaly. These symptoms can include urinary incontinence, alterations in mental status, memory problems, weakness, and ataxia of the lower extremities, as was seen in the case presented [4]. Alterations in mental status can present as a schizophrenia-like psychosis [6-7]. New-onset seizure activity has been described as a presentation of colloid cysts of the third ventricle [8].In children, the most common symptoms are headaches associated with nausea and vomiting [9].

Acute deterioration and sudden death have been described in the literature. Purported causes include not only the ball-valve mechanism hypothesis but also direct stimulation of the hypothalamus from colloid cyst compression [10]. Acute brain herniation has also been proposed as a cause of sudden death in patients with colloid cysts of the third ventricle [11]. Physical exertion has been associated with acute presentations of a colloid cyst of the third ventricle [3]. The patient presented was climbing stairs, which is a form of exertion.

Physical examination findings are often nonspecific. Papilledema may be seen at the time of diagnosis. Ataxia may be found. In children, diplopia as well as papilledema may be present [2]. Colloid cysts in children are more likely to be detected as incidental findings, have lower rates of associated hydrocephalus, and are less likely to show acute deterioration in clinical status [9].

Work-up

Computerized tomography (CT) or magnetic resonance imaging (MRI) can diagnose a colloid cyst. The initial imaging study of choice is a CT scan of the head without contrast [4]. The characteristic CT finding is an ovoid lesion within the third ventricle, which can be of varying density. The lesion may be enhanced with contrast [1].

On MRI, colloid cysts are visualized as hyperintense on T1-weighted images and hypointense on T2-weighted images [1]. It is important to remember that lumbar puncture in the context of obstructive hydrocephalus can lead to herniation [4].

Treatment

Ventricular shunting may be needed prior to definitive surgery in the acute management of colloid cysts [1]. Multiple surgical approaches have been described, including transcortical surgery or endoscopic removal [1]. Recurrence after resection is rare [1].

## Conclusions

Although histologically benign, colloid cysts can present with significant neurological sequelae and associated morbidity and mortality. The most common symptom is headache, which can be sudden, brief, or intense. Patients can present with a variety of neurologic symptoms, including urinary incontinence, alterations in mental status, and memory problems. Weakness and ataxia of the lower extremities can occur. In children, the most common symptoms are headaches associated with nausea and vomiting. The patient in this case presented with a headache and transient lower extremity weakness. Her MRI showed a 9-mm colloid cyst at the foramen of Monro, causing an obstructive hydrocephalus that required surgical removal. The goal of this case report is to familiarize readers with the varied presentations, symptomatology, and management associated with a colloid cyst.
